# Decreasing mortality and hospitalizations with rising costs related to gastric cancer in the USA: an epidemiological perspective

**DOI:** 10.1186/s13045-018-0682-5

**Published:** 2018-12-13

**Authors:** Delong Liu, Dhruv Mehta, Supreet Kaur, Arun Kumar, Kaushal Parikh, Lavneet Chawla, Shanti Patel, Amirta Devi, Aparna Saha

**Affiliations:** 1 Department of Oncology, The First affiliated Hospital of Zhengzhou University, Zhengzhou, 450052 China; 2 New York Medical College and Westchester Medical Center, Valhalla, NY USA; 3 Department of Hematology and Oncology, St Joseph’s Regional Medical Center, Patterson, NJ USA; 4 Department of Internal Medicine, Maimonides Medical Center, Valhalla, NY USA; 5 Dow University of Health Sciences, Karachi, Pakistan; 6 Department of Nephrology, Icahn School of Medicine, New York, NY USA

**Keywords:** Inpatient admission rates, Gastric cancer, Epidemiology, National inpatient database

## Abstract

**Background:**

There is no convincing data on the trends of hospitalizations, mortality, cost, and demographic variations associated with inpatient admissions for gastric cancer in the USA. The aim of this study was to use a national database of US hospitals to evaluate the trends associated with gastric cancer.

**Methods:**

We analyzed the National Inpatient Sample (NIS) database for all patients in whom gastric cancer (ICD-9 code: 151.0, 151.1, 151.2, 151.3, 151.4, 151.5, 151.6, 151.8, 151.9) was the principal discharge diagnosis during the period, 2003–2014. The NIS is the largest publicly available all-payer inpatient care database in the US. It contains data from approximately eight million hospital stays each year. The statistical significance of the difference in the number of hospital discharges, length of stay, and hospital costs over the study period was determined by regression analysis.

**Results:**

In 2003, there were 23,921 admissions with a principal discharge diagnosis of gastric cancer as compared to 21,540 in 2014 (*P* < 0.01). The mean length of stay for gastric cancer decreased by 17% between 2003 and 2014 from 10.9 days to 8.95 days (*P* < 0.01). However, during this period, the mean hospital charges increased significantly by 21% from $ 75,341 per patient in 2003 to $ 91,385 per patient in 2014 (*P* < 0.001). There was a more significant reduction in mortality over a period of 11 years from 2428 (10.15%) in 2003 to 1345 (6.24%) in 2014 (*P* < 0.01). The aggregate charges (i.e., “national bill”) for gastric cancer increased significantly from 1.79 bn $ to 1. 96 bn $ (*P* < 0.001), despite decrease in hospitalization (inflation adjusted).

**Conclusion:**

Although the number of inpatient admissions for gastric cancer have decreased over the past decade, the healthcare burden and cost related to it has increased significantly. Inpatient mortality is decreasing which is consistent with overall decrease in gastric cancer-related deaths. Cost increase associated with gastric cancer contributed significantly to the national healthcare bill.

## Introduction

According to the World Health Organization (WHO) International agency for research on cancer, approximately one million new cases of gastric cancer were reported in 2012, making it the fifth most common cancer in the world, after lung, breast, colorectal, and prostate cancer [1]. Over the past few years, a steady decline is seen in the age-adjusted gastric cancer incidence rate in the developed countries including North America and Europe [2–6]. These trends can be attributed in part to lifestyle and hygiene changes such as a reduced consumption of foods preserved by salting or smoking, and a reduction in the prevalence of Helicobacter pylori (*H. pylori*) infection [7, 8]. Gastric cancer carries a rather poor prognosis, with cohort and period estimate of 5-year relative survival rate below 25% [9, 10]. The most common treatment option for gastric cancers is surgery with or without perioperative chemotherapy for localized disease and chemotherapy alone for metastatic disease. Immune checkpoint inhibitors have been approved for treatment of several solid tumors and recently become a major option for gastric cancer therapy [11–16]. Cancer immunotherapy including chimeric antigen receptor T cells and bispecific antibodies are rapidly changing the landscape of therapeutic approaches for solid tumors including gastric cancers [17–24].

Despite a decline in gastric cancer incidence, it remains one of the most common causes of cancer-related morbidity and mortality and has a significant impact on the United States (US) healthcare system [25, 26]. To the best of our knowledge, there are no reported studies to assess the demographic variations and trends of inpatient hospitalization in gastric cancer in the US population. This study is conducted to delineate the inpatient trends of hospitalization, mortality, length of stay, cost, and demographic variations of gastric cancer-related admissions in the USA from the period of 2003 to 2014. The study found that the number of inpatient admissions for gastric cancer has decreased over the past decade, though the healthcare burden and cost related to it have increased significantly. Inpatient mortality is decreasing which is consistent with overall decrease in gastric cancer-related deaths.

## Research design and methods

### Research database

We obtained a population-based estimate of inpatient hospitalization utilizing National Inpatient Sample (NIS), developed by the agency for Healthcare Research and Quality (AHRQ, Rockville, Maryland), as a part of Healthcare Cost and Utilization Project (HCUP) through a Federal-State-Industry partnership. The details of this database has been described [27, 28].

The NIS contains approximately 20% stratified sample of discharges from community (non-federal) hospitals and is the largest publicly available all-payer inpatient health care data set in the USA. The 2003 NIS data were selected using a stratified probability sample of hospitals, drawn from a frame of 37 states containing approximately 8.0 million discharges from 994 hospitals, with weights to facilitate national estimates. The 2014 NIS sampling frame is obtained from more than 4400 hospitals in 44 states and the District of Columbia and contains approximately seven million discharges. The NIS is an excellent representative sample of the general US inpatient population, representing > 95% of the US population and provides a comprehensive database for analyzing healthcare utilization, access, charges, quality, and outcomes. This database provides only administrative data for analysis. Patient-specific clinical data is lacking.

We identified cases of gastric cancer by querying the NIS database for hospital data on all discharge diagnoses with a primary ICD-9 CM diagnosis code of 151 (151.0, 151.1, 151.2, 151.3, 151.4, 151.5, 151.6, 151.8, 151.9) from 2003 to 2014.

### Patients and hospitals

Patient demographics, including age, sex, and insurance status, was obtained from the NIS database. Various hospital characteristics, including location (Northeast, Midwest, South and West and metropolitan vs non-metropolitan area), type (teaching vs non-teaching), and size (small, medium, and large) were recorded. Metropolitan areas were defined as those with a population of at least 50,000 people. A teaching hospital was defined as one designated as an American Medical Association (AMA)-approved residency program by the American Hospital Association Annual Survey, a member of the Council of Teaching Hospitals or has a ratio of full-time equivalent interns and residents to beds of 0.25 or higher. The definition of bed-size varied according to hospital location and teaching status and hence there is a large overlap in the definition of hospital size. For small hospitals, bed-size ranged from 1 to 299 beds, for medium hospitals the range was between 50 and 499, and for large hospitals it was between 100 and 500 beds or higher (Appendix). We also obtained the payer status for all admissions. “Length of stay” was defined as the number of nights the patient remained in the hospital for this inpatient visit.

### Statistical analysis

The trends for the annual point estimates of frequency of gastric cancer for the sample were analyzed. The annual frequency of discharges with a diagnosis of gastric cancer was computed by dividing the annual number of discharges with gastric cancer listed in the NIS database in each year by the total number of all discharges listed in the NIS for the same year. The temporal trend in frequencies of discharges, lengths of stay, hospital charges, and frequencies of deaths in patients with gastric cancer was estimated by linear and polynomial regression. The most appropriate functional form for the trend was assessed by examination of regression diagnostic plots. Linear shape was determined for hospital charges and in-hospital deaths: a quadratic shape for length of stay and a cubic shape for number of discharges and discharge rate. A *P* value < 0.05 was considered statistically significant. All analyses were performed using SAS (version 9.4, The SAS Institute, Cary, NC).

In addition to the percentages available adjacent to the data in the tables, the frequency per 10,000 admissions were also calculated for each categorical variable. These numbers represent the density of patients diagnosed with gastric cancer compared with the total number of hospital discharges per category. Each frequency was calculated by dividing the number of patients with a diagnosis of gastric cancer by the total discharges in a specific categorical variable for each year and multiplying that number by 10,000. We viewed the counts as arising from a Poisson distribution and the total discharges as an offset, yielding Poisson rates that were compared over time using Poisson regression and yielded relative rates (RRs) and 95% confidence intervals (CIs) that expressed the ratio of rate per 10,000 in 2014 to that of 2003. These values differed from the percentages, which describe each category exclusively for either patients with gastric cancer or for total discharges. The percentages distinguished differences among the variables for each specific year, whereas the frequencies were vital for comparing trends from 2003 to 2014, especially for age group and region.

## Results

### Number and costs of gastric cancer discharges

**Fig. 1:**
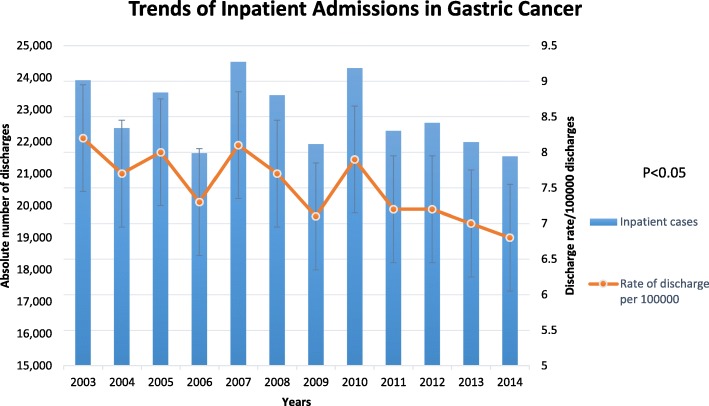
Trends of gastric cancer hospitalizations

The absolute number of admissions for gastric cancer as the primary diagnosis showed a decreasing trend from 23,921 in 2003 to 21,540 in 2014 (*P* < 0.01). Expectedly, discharges with gastric cancer also decreased from 6.45 per 10,000 discharges in 2003 to 6.09 per 10,000 discharges in 2014 (RR 0.94, 95% CI 0.92–0.96, *P* < 0.0001) (Fig. 1).

**Fig. 2:**
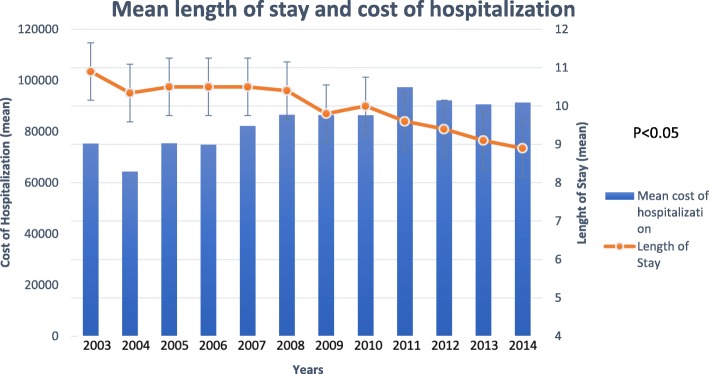
Mean length of stay and cost of hospitalization

The average length of hospital stay for patients with gastric cancer as a principal diagnosis decreased from 10.9 days in 2003 to 8.95 days in 2014 (*P* < 0.01). Despite the decrease in the average length of hospital stay, the mean total charges for gastric cancer-related hospital admissions showed a considerable increase between 2003 and 2014. After adjusting for inflation, mean hospital charges per patient increased by 21.2% in a statistically significant linear fashion from $75,341 in 2003 to $91,385 in 2014 (*P* < 0.001). The total aggregate cost “national burden” for hospitalizations with gastric cancer as the discharge diagnosis increased from $1.79 billion in 2003 to $1.96 billion in 2014 (inflation adjusted) (*P* < 0.001) (Fig. 2).

### Reduction in mortality

**Fig. 3:**
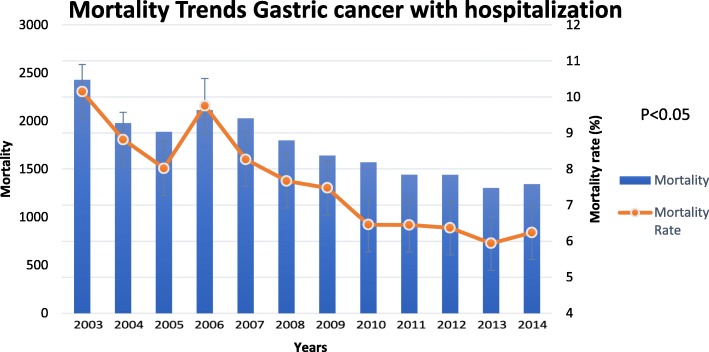
Mortality trends in gastric cancer with hospitalization

Although there was a slight decrease in the frequency of hospital discharges from 2003 to 2014, there was a more significant reduction in mortality over a period of 11 years from 10.15% in 2003 to 6.24% in 2014 (*P* < 0.01) (Fig. 3).

### Patient characteristics by age

The highest rate of discharges with the principal diagnosis of gastric cancer was seen in age group of 65–84 years. There was 12% decrease in the frequency of discharge rates in the age group of 65–84 years from 12,420 in 2003 to 10,255 in 2014 (RR = 0.88 95% CI 0.86–0.90; *P* < 0.0001). The frequency of discharge rates was very similar in the age group of 45–64 years without any statistical significance. The frequency of discharges showed a decline from 9.6 per 10,000 admissions in 2003 to 6.6 per 10,000 admissions in 2014 in the + 85-age group (RR = .68, 95% CI 0.64–0.73, *P* < 0.0001). Interestingly, the only group that showed an increment in the frequency of hospitalization was the age group 18–44 years from 1.66 per 10,000 admissions in 2003 to 1.82 per 10,000 admissions in 2014 (RR = 1.1, 95% CI 1.02–1.17; *P* < 0.05) (Table 1).

**Table 1:** Sociodemographic and patient characteristics related to primary diagnosis of gastric cancer

	Categorical variable	2003	2014	2003	2014	Gastric cancer per 10,000 admissions in 2003	Gastric cancer per 10,000 admissions in 2014	RR (95% CI), *P* value
		Gastric cancer	Gastric cancer	(*N*, %)	(*N*, %)			
		(*N*, %)	(*N*, %)					
All discharges		23,921 (100.00%)	21,540 (100%)	37,074,605 (100.00%)	35,358,818 (100.00%)	6.45	6.09	0.94 (0.92–0.96) *P* < 0.0001
Mean age (years)	18–44	1630 (6.82%)	1595 (7.40%)	9,772,014 (26.36%)	8,714,895 (24.65%)	1.66	1.83	1.1 (1.02–1.17); *P* = 0.008
	45–64	7291 (30.48%)	7795 (36.19%)	8,086,876 (21.81%)	8,709,298 (24.63%)	9.01	8.95	0.99 (0.96–1.02); *P* = 0.6
	65–84	12,420 (51.92%)	10,255 (47.61%)	10,150,753 (27.38%)	9,490,054 (26.84%)	12.2	10.8	0.88 (0.86–0.9); *P* < 0.0001
	85+	2569 (10.74%)	1880 (8.73%)	2,666,613 (7.19%)	2,837,716 (8.03%)	9.6	6.6	0.68 (0.64–0.73); *P* < 0.0001
Sex	Male	14,916 (62.36%)	14,045 (65.20%)	15,064,915 (40.63%)	15,095,708 (42.69%)	9.9	9.3	0.93 (0.91–0.96); *P* < 0.0001
	Female	8979 (37.54%)	7470 (34.68%)	21,861,583 (58.97%)	20,255,555 (57.29%)	4.1	3.68	0.89 (0.87–0.92); *P* < 0.0001
Payer	Medicare	13,549 (56.64%)	11,210 (52.04%)	13,761,829 (37.12%)	13,795,116 (39.01%)	9.8	8.1	0.82 (0.8–0.84); *P* < 0.0001
	Medicaid	1917 (8.01%)	2780 (12.91%)	6,828,282 (18.42%)	7,993,545 (22.61%)	2.8	3.47	1.2 (1.1–1.3); *P* < 0.0001
	Private insurance	7009 (29.30%)	6055 (28.11%)	13,555,962 (36.56%)	10,833,048 (30.64%)	5.17	5.5	1.08 (1.04–1.12); *P* < 0.001
	Uninsured	841 (3.52%)	855 (3.97%)	1,707,382 (4.61%)	1,650,461 (4.67%)	4.9	5.1	1.05 (0.95–1.1); *P* = 0.29
	Other	578 (2.42%)	595 (2.76%)	1,147,219 (3.09%	1,019,269 (2.88%)	5.0	5.8	1.15 (1.03–1.2); *P* = 0.01
Median income	Low ($0–35,999)	6042 (25.26%)	5835 (27.09%)	10,061,048 (27.14%)	10,244,655 (28.97%)	6	5.7	0.94 (0.91–0.98); *P* = 0.003
	Not low ($36,000+)	17,389 (72.69%)	15,190 (70.52%)	26,173,832 (70.60%	24,344,858 (68.85%)	6.6	6.2	0.93 (0.91–0.95); *P* < 0.0001
	Missing	490 (2.05%)	515 (2.39%)	839,725 (2.26%	769,305 (2.18%)	5.8	6.7	1.1 (1.01–1.29); *P* = 0.02
Hospital owner	Government	3091 (12.92%)	3075 (14.28%	5,172,217 (13.95%	4,310,458 (12.19%)	5.97	7.13	1.2(1.13–1.25); *P* < 0.0001
	Private, not-for-profit	18,042 (75.42%)	16,150 (74.98%)	26,964,496 (72.73%	25,831,562 (73.06%)	6.7	6.25	0.93(0.91–0.95); *P* < 0.0001
	Private, for-profit	2788 (11.66%)	2315 (10.75%)	4,937,891 (13.32%)	5,216,798 (14.75%)	5.65	4.4	0.78 (0.74–0.83); *P* < 0.0001
Location	Rural	2331 (9.74%)	915 (4.25%)	5,583,485 (15.06)	3,360,976 (9.51%)	4.2	2.7	0.65 (0.6–0.7); *P* < 0.0001
	Urban	21,590 (90.26%)	20,625 (95.75%)	31,471,911 (84.89)	31,997,842 (90.49%)	6.9	6.45	0.93 (0.92–0.95); *P* < 0.0001
Bed size	Small	2320 (9.70%)	3135 (14.55%)	4,327,304 (11.67%)	6,553,063 (18.53%)	5.4	4.8	0.9 (0.84–0.94); *P* < 0.0001
	Medium	5577 (23.31%	5310 (24.65%)	9,613,451 (25.93%)	10,398,925 (29.41%)	5.8	5.1	0.88 (0.84–0.91); *P* < 0.0001
	Large	16,024 (66.99%)	13,095 (60.79%)	23,114,641 (62.35%)	18,406,830 (52.06%)	6.9	7.1	1.02 (1.002–1.005) *P* = 0.02
Region	Northeast	5300 (22.16%)	4830 (22.42%)	7,264,150 (19.59%)	6,623,697 (18.73%)	7.3	7.3	0.99 (0.96–1.06); *P* = 0.9
	Midwest	4654 (19.45%)	4430 (20.57%)	8,520,023 (22.98%)	7,942,913 (22.46%)	5.4	5.57	1.02 (0.97–1.06); *P* = 0.3
	South	8292 (34.66%)	7755 (36.00%)	14,205,434 (38.32%)	13,774,248 (38.96%)	5.8	5.6	0.96 (0.93–0.98); *P* = 0.02
	West	5676 (23.73%)	4525 (21.01%)	7,084,998 (19.11%)	7,017,960 (19.85%)	8.0	6.4	0.8 (0.77–0.83); *P* < 0.0001

### Patient characteristics by sex

The frequency of gastric cancer discharges was higher in males; however, the decrease in the frequency of gastric cancer-related hospitalization from 2003 to 2014 was slightly more prominent in females. Male hospitalization in both 2003 and 2014 were 2.4-folds higher than that of their female counterparts. The frequency of discharges in men decreased from 9.9 per 10,000 admissions in 2003 to 9.3 per 10,000 admissions in 2014 (RR = 0.93, 95% CI 0.91–0.96; *P* < 0.0001). The frequency of discharges in women decreased from 4.1 per 10,000 admissions in 2003 to 3.68 per 10,000 admissions in 2014 (RR = 0.89, 95% CI 0.87–0.92; *P* < 0.0001). (Table 1).

### Patient characteristics by payer group and income

Between 2003 and 2014, the relative frequency of gastric cancer discharges decreased for Medicare users and increased for Medicaid and private insurers. In both 2003 and 2014, the highest absolute number of discharges were noted in the Medicare group (56.6% in 2003 and 52.04% in 2014). The decrease in the relative frequency of gastric cancer discharges was seen to be greatest in Medicare patients. The discharge rate decreased from 9.8 per 10,000 admissions in 2003 to 8.1 per 10,000 admissions in 2014 (RR = 0.82, 95% CI 0.80–0.84, *P* < 0.0001). Medicaid group showed an increase in the relative frequency of discharges by 20% from 2.8 per 10,000 admissions in 2003 to 3.47 per 10,000 admissions in 2014 (RR = 1.2, 95% CI 1.1–1.3; *P* < 0.0001), followed by private insurance (5.17 per 10,000 in 2003 and 5.5 per 10,000 in 2014 with RR = 1.08) with no changes observed in uninsured group.

Patients were categorized into low and not-low income groups based on median income for zip code. In both 2003 and 2014, nearly 70% of the absolute number of discharges were considered in the “not-low” income group. Both groups showed similar decrement of around 7% in the number of discharges over the study period (*P* < 0.01) (Table 1).

### Patient discharges by hospital characteristics and region

During 2003 and 2014, metropolitan areas had higher absolute number as well as frequency of gastric cancer discharges than those from the non-metropolitan areas. In metropolitan areas, the frequency of discharges decreased from 6.9 per 10,000 in 2003 to 6.45 in 2014 (RR = 0.93, 95% CI 0.92–0.95, *P* < 0.0001). The frequency of discharges in non-metropolitan areas showed a more dramatic decrease from 4.2 per 10,000 to 2.7 per 10,000 (RR = 0.65, 95% CI 0.6–0.7, *P* < 0.0001).

Between 2003 and 2014, the relative frequency of gastric cancer discharges decreased for small and medium size hospitals and slightly increased for large size hospital (*P* < 0.01). In both 2003 and 2014, the highest absolute number of discharges were noted in the large size hospital group (66.99% in 2003 and 60.79% in 2014).

The South had the highest number of gastric cancer discharges during 2003 and 2014 (34.66% and 36% respectively). The highest relative frequency of discharges was seen in the west at 8 per 10,000 admissions in 2003 and northeast with 7.3 per 10,000 admissions in 2014. The change in the relative frequency of discharges for northeast and midwest regions remained statistically insignificant from 2003 to 2014. The frequency of discharges in the south decreased from 5.8 per 10,000 in 2003 to 5.6 per 10,000 in 2014 (RR = 0.96, 95% CI 0.93–0.98; *P* < 0.05). The frequency of discharges for the west showed a 20% decrease in the rate of hospitalization from 8 per 10,000 in 2003 to 6.4 per 10,000 in 2014 (RR = 0.80, 95% CI 0.77–0.83; *P* < 0.001). (Table 1)

## Discussion

We studied the trends of inpatient hospitalizations, mortality, and costs in patients with gastric cancer during the period from 2003 to 2014. We found a statistically significant decline in gastric cancer hospitalizations during the study period, which can be explained by an overall decline in gastric cancer incidence and advances in outpatient management of these patients, leading to fewer admissions [29, 30]. *H. pylori* infection plays a central role in the development of gastric cancer and a decline in incidence rates of gastric cancer correlated to an overall decline in and improved management of *H. pylori* infection [31, 32]. This trend may also be related to improvements in living conditions, water supply, drainage, and better hygiene related to food consumption [33]. Studies have shown an increase in adoption of novel and evidence-based gastric cancer therapies, such as endoscopic resection of early cancer and use of adjuvant chemotherapy in locally advanced disease which are associated with improved survival in early-stage cancers and can explain lesser numbers of unplanned admissions [34]. With earlier and increasing use of immune checkpoint inhibitors for therapy of gastric cancer, further decline in admission and hospitalization for gastric cancer is expected [34]. Future analysis of discharge data from NIS will be able to provide evidence on this.

Our study showed that the highest inpatient hospitalizations secondary to gastric cancer are seen in elderly patients, which can be explained by the higher incidence of gastric cancer in the elderly patients above age 65. Elderly patients may often have comorbidities which increase the 30-day postoperative mortality rates and some of these patients may not be considered fit for curative resection even in early disease secondary to significant comorbidities [35]. Moreover, studies have also shown that elderly patients often have a more advanced stage of the non-cardia gastric cancers, a higher incidence of cardia cancers, which are associated with a poor prognosis [36–38]. The major treatment option for the advanced stages of gastric cancer used to be chemotherapy. The palliative chemotherapy carries a poor response rate and multiple complications secondary to therapy or the cancer, such as gastrointestinal (GI) bleeding, gastric outlet/intestinal obstruction, peritoneal carcinomatosis and intractable vomiting, which result in poor quality of life and multiple hospitalizations in these patients [39]. Interestingly, we found there was a decrease in hospitalization trends in all age groups except in younger patients age 18–44 years, which showed an increment in inpatient admissions. We postulate that the younger patients have lesser number of comorbidities and are more amenable to curative resections and early aggressive therapies requiring frequent inpatient hospitalizations. There is an improved overall survival in gastric cancers, especially in younger patients [40]. However, we postulate that the morbidity associated with gastric cancer including complications secondary to chemotherapy and/or surgical interventions requires multiple hospitalizations. Overall, we found a decline in gastric cancer-related hospitalizations in other age groups, which coincides with an overall decline in gastric cancer incidence. This study also found an increased number of discharges from the metropolitan hospitals as compared to non-metropolitan hospitals. This may be explained by a multidisciplinary approach in the metropolitan hospitals towards the care of the gastric cancer patients and early involvement of the palliative care team, especially in patients with advanced disease, thus leading to decreased hospital mortality, better quality of life, lesser number of hospitalizations, and higher rates of discharges to hospice care [41]. Furthermore, low socioeconomic status is one of the factors prevalent in patients with gastric cancer, leading to the highest number of admissions and discharges in the government hospitals [42].

We also found a decline in gastric cancer-related inpatient mortalities during the study period, which can be related to the improved management of gastric cancers and overall improved survival with the advent of more sophisticated surgical techniques and use of preoperative chemotherapies [43]. Furthermore, the implementation of hospice care for patients with advanced disease unamenable to aggressive therapy has decreased the rate of inpatient hospitalizations and overall inpatient deaths [44]. Terminal patients with hospice care require less admissions to acute care wards and intensive care units and have improved quality of life [45].

The average length of hospital stay has shortened during the study period from 10.9 days in 2003 to 8.95 days in 2014. It can be related to better management of chemotherapy-related complications and more sophisticated surgical techniques causing lesser complications resulting in early discharges. However, we found a significant increase in cost of hospitalizations with gastric cancer. It is likely related to the (1) increasing overall cost of health care, particularly the higher cost of targeted agents, (2) cost of palliation- interventions for pain management and palliative alleviation of symptoms, and (3) use of palliative chemotherapy as standard of care in advance-stage gastric cancer and costs associated with the management of chemotherapy complications and morbidity secondary to advanced cancer [46]. Per the NIS database, the mean cost of all hospitalization increased by 16.2% from 2003 to 2014 (9364$ to 10,885$ after adjusting for inflation). With gastric cancer, an increase of about 21.2% was noted during the same period. This can be attributed to the increasing cost of hospitalization as well as more health care dollar spent on gastric cancer care ($75,341 in 2003 to $91,385 in 2014).

Most patients with gastric cancer are elderly with access to Medicare insurance, which explains the highest absolute number of admissions in Medicare group. In general, we found disparities in the gastric cancer hospitalization depending upon insurance type. Uninsured patients or patients with Medicare or Medicaid are known to have lower odds of having recommended outpatient follow-ups and receiving chemotherapy or radiation therapy, resulting in higher incidence of emergency department visits and hospitalizations [47]. Studies have also shown racial/ethnic disparities seen in the use of evidence-based therapies including preoperative chemotherapy and radiation therapy, which result in disparities in overall survival and advanced cancer and chemotherapy complications requiring multiple hospitalizations [48]. We also found a significant difference in gastric cancer-related hospitalizations secondary to gender, with higher number of admissions in males than females. This correlates with higher incidence of gastric cancer in males [49]. While the mechanisms leading to gender disparity are unconfirmed, studies have linked genetic mutations in males, specifically disruption of androgen receptor (AR) homeostasis, and other lifestyle-related risk factors, such as higher consumption of fried food and smoking, with development of gastric cancer [49, 50].

NIS data set is purely administrative and is very dependent on documentation and coding practices of healthcare institutions and individual physicians. The nature of the NIS data set and the study design are the first and foremost limitations of our study. It is most likely that our study underestimates the actual incidence of gastric cancer-related hospitalizations, as patient admissions may have been coded with other primary diagnosis such as chemotherapy complications or complications of advanced disease such as intestinal obstruction and GI bleeding. Other possible limitations include any errors that may have occurred during data entry and inability to obtain individual patient-specific clinical information, such as race/ethnicity, laboratory values, and procedures performed, thereby limiting observations to the given demographics of the study sample. Lastly, NIS data set only provided limited data regarding the hospital variables and does not provide sufficient patient and hospital details to determine the factors that could potentially explain the significant decline in hospital discharges and a rise in cost of care.

In conclusion, gastric cancer-related hospitalizations and mortality have declined over the last decade. However, it remains a growing concern in the US healthcare system secondary to an increase in health care costs and burden. Despite a decline in gastric cancer-related hospitalization and mortality, the overall cost of care continues to rise in the last decade. Further studies are warranted on cost-effective evaluation and management of gastric cancer.

## Appendix

**Table 2:** NIS description of hospital bedsize

Bedsize categories			
Location and teaching status	Hospital bedsize		
	Small	Medium	Large
Northeast region			
Rural	1–49	50–99	100+
Urban, non-teaching	1–124	125–199	200+
Urban, teaching	1–249	250–424	425+
Midwest region			
Rural	1–29	30–49	50+
Urban, non-teaching	1–74	75–174	175+
Urban, teaching	1–249	250–374	375+
Southern region			
Rural	1–39	40–74	75+
Urban, non-teaching	1–99	100–199	200+
Urban, teaching	1–249	250–449	450+
Western region			
Rural	1–24	25–44	45+
Urban, non-teaching	1–99	100–174	175+
Urban, teaching	1–199	200–324	325+

## Abbreviations

HCUP: Healthcare Cost and Utilization Project
NIS: National Inpatient Sample
